# Effects of a Community-Based Program on Voluntary Modern Contraceptive Uptake Among Young First-Time Parents in Cross River State, Nigeria

**DOI:** 10.9745/GHSP-D-20-00111

**Published:** 2020-12-23

**Authors:** Gwendolyn Morgan, Anjala Kanesathasan, Akinsewa Akiode

**Affiliations:** a International Business & Technical Consultants, Inc., Vienna, VA, USA.; b Independent consultant, Washington, DC, USA.; c Research & Communication Services, Abuja, Nigeria.

## Abstract

**Background:**

Reproductive health programs for youth have largely overlooked first-time parents (FTPs)—defined as young women younger than 25 years old who are pregnant or already have 1 child, and their partners. To address this gap, we implemented and evaluated a program to improve child spacing, modern contraceptive use, and related gender outcomes among FTPs in Cross River State (CRS), Nigeria. This paper examines the effectiveness of FTP interventions in improving voluntary uptake of contraception.

**Methods:**

We conducted small group sessions and home visits with FTPs from May to August 2018 in 2 local government areas of CRS. A pretest–posttest study examined the effectiveness of these interventions regarding healthy timing and spacing of pregnancy/family planning knowledge, attitudes, intentions, communication, decision making, and contraceptive use. We performed a bivariate analysis and logistic binomial regression to confirm change over time in the primary study outcome, current use of a modern method of contraception. We also performed analysis of demographic characteristics and secondary outcomes (e.g., birth spacing intentions and couple communication).

**Results:**

We interviewed 338 participating first-time mothers (FTMs) and 224 participating partners at baseline and endline. Important indicators of contraceptive awareness, attitudes, and couples’ communication increased significantly from baseline to endline. Voluntary current modern contraceptive use increased from 26% to 79% among nonpregnant FTMs (*P*<.000), and from 44% to 81% among partners (*P*<.000). After controlling for significant factors related to family planning use, FTMs were 3.3 times more likely (*P*<.001) and partners 3.7 times more likely (*P*<.000) to be using a modern contraceptive method at endline.

**Conclusion:**

Program participation was associated with significant improvements in voluntary uptake of modern contraceptive methods and multiple secondary outcomes. Even within a short timeframe, this intensive, multi-intervention effort achieved significant advancements across healthy timing and spacing of pregnancy and family planning outcomes for this vulnerable youth population.

## Abstract (precis)

Among young first-time mothers, participation in a comprehensive, community-based program led to a 3-fold increase in voluntary modern contraceptive use and other positive changes. These results demonstrate the importance of investing in interventions for this youth population that ideally address a range of priorities across the first-time parent lifestage.

## Teaser

**Key Findings:**

We implemented and evaluated a program to improve child spacing, modern contraceptive use, and related gender outcomes among first-time parents in Cross River State, Nigeria.

- Contraceptive awareness, attitudes, and couples’ communication increased significantly from baseline to endline.
- After controlling for significant factors related to family planning use, first-time mothers were 3.3 times more likely and male partners 3.7 times more likely to be using a modern contraceptive method at endline. Most first-time mothers and their partners preferred the contraceptive implant, and a smaller percentage chose the injectable.

**Key Implications:**

This experience suggests that local and state governments can adapt and scale up 3 essential program elements:

- Ensure the availability of modern contraceptive methods through local health facilities
- Use community-based health workers to conduct home visits with first-time parents to provide tailored health information and referrals, as well as build linkages with the formal health sector
- Include activities that address gender norms and couple dynamics to foster better alignment, communication, and joint action on reproductive issues

## INTRODUCTION

Longer birth intervals, facilitated by modern contraceptive use, are associated with reductions in maternal and neonatal mortality and morbidity in low- and middle-income countries.^1^ ^,^ ^2^ Adolescent mothers around the world disproportionately experience pregnancy-related death and disease when they start childbearing early and have rapid repeat pregnancies. Global studies show that adolescents aged 15–19 years have less access to voluntary modern contraception, use modern contraceptives less frequently, and have a higher unmet need for modern contraception than older women.^3^ ^–^ ^5^ These factors place adolescent and young mothers at risk of negative health outcomes^6^ and highlight the particular vulnerabilities that young women and mothers face, including those going through pregnancy, childbirth, and childrearing for the first time.

This global pattern is reflected in Cross River State (CRS), Nigeria. As of May 2017, available national- and state-level data showed that sexual activity and motherhood began early. In CRS, 18% of adolescent girls aged 15–19 years have started childbearing.^7^ In addition, adolescent and young mothers often do not use modern contraception to space their second child or subsequent children. As a result, rapid repeat pregnancies are common, with nearly one-quarter of all children born less than 2 years after a sibling.^7^ Only 27% of sexually active adolescent girls (15–19 years) in CRS reported using a modern contraceptive method (both married and unmarried).^7^

As noted in a 2007 literature review by the World Health Organization^8^, the social and economic consequences of adolescent sexuality and pregnancy greatly depend on an adolescent’s particular cultural, family, and community setting. In CRS (as in other parts of Nigeria and in the global context), where fertility is highly valued within the institution of marriage, unmarried young mothers often endure additional stigma and discrimination within their communities and families due to an early, unplanned, and unwanted pregnancy.^9^ ^–^ ^11^ As a result, they may experience adverse social consequences such as curtailment of their education, decreased mobility, financial deprivation, and increased social isolation.^9^ ^,^ ^12^ Young men who unexpectantly become fathers for the first time may also face numerous challenges in continuing their education and providing adequate financial support for themselves and their new family.^9^ ^,^ ^13^

Adolescent and young mothers also face critical barriers (financial, physical access, family permission, etc.) in seeking health care and services.^8^ Key influencers, such as parents, in-laws, husbands or male partners, and perhaps older co-wives, typically drive household decision making as well as health care spending. Evidence shows that couples who discuss and jointly make decisions about family planning and use of contraception, as well as receive social support for using contraceptive methods and services, are more likely to use contraception.^14^ ^–^ ^16^ Men who approve of their female partners’ contraceptive use, provide support to obtain transport to reach a facility, and provide funds and permission to access services are critical to facilitating women’s contraceptive use in many country contexts, including Nigeria.^17^ ^,^ ^18^ Yet male partners, parents, and even in-laws may implicitly or explicitly discourage use of contraception due to concerns about perceived and actual side effects. They may also simply fail to give permission to visit a health facility or give financial support for these services to new young mothers, especially those who are not yet empowered to initiate conversations about family planning.

In addition, adolescents and young women themselves have their own biases and misinformation about the risks and potential side effects of contraception, such as infertility, permanent damage to reproductive organs, infections, or cancer.^19^ ^,^ ^20^ Methods that interrupt the perceived natural pattern of menstruation are largely deemed unacceptable. Adolescents and young women often perceive that they will be more likely to experience these side effects if they use long-acting contraception, such as an intrauterine device or implant. Improving the attitudes of adolescent and young mothers and their partners about the healthy timing and spacing of pregnancies (HTSP) and supporting their informed knowledge and voluntary use of modern contraception are particular priorities for health programs, given the higher risks of morbidity and mortality for both the mother and the child.^21^

First-time parents (FTPs)—defined as young women under age 25 who are pregnant or already have 1 child, and their partners—have largely been overlooked in reproductive health programs for youth. A 2014 review of global data showed that many first-time mothers (FTMs) are at increased risk of poor pregnancy, delivery, and child health outcomes, a situation compounded by multiple factors that limit their access to timely health information and services.^22^ The needs of FTPs extend beyond the scope of many adolescent and youth programs, which often cater to unmarried clients and focus on the prevention of pregnancy. Issues faced by young parents, such as infant care and feeding or couple communication and decision making are also not typically included in family planning and pregnancy prevention programs aimed at women of reproductive age or even married youth.

First-time parents have largely been overlooked in reproductive health programs for youth.

To address this gap in CRS, the Evidence to Action (E2A) Project, a global family planning project funded by United States Agency for International Development, and Pathfinder International/Nigeria launched a program to improve child spacing, voluntary contraceptive use, and related gender outcomes among FTMs and their male partners. Implemented through the Saving Mothers, Giving Life (SMGL) Initiative, and in partnership with the CRS Ministry of Health, the program focused on increasing access to HTSP and family planning information and services, as well as addressing the underlying social and gender factors that influence family planning communication, decision making, and action for FTMs and their male partners. The program applied both a life course and a socioecological lens to determine the appropriate content and structure of interventions with young FTMs, their husbands/partners, other key influencers, and the broader community.^23^ We also built on existing facility- and community-based family planning services, strengthened under the ongoing SMGL Initiative, to provide targeted family planning counseling and referral linkages for FTMs and their male partners. The package of FTP interventions was implemented in 2 local government areas (LGAs) of CRS, Ikom and Obubra, from May through August 2018.

This article examines the effectiveness of community-based FTP interventions in improving FTPs’ demand for HTSP and their voluntary uptake of contraception through analysis of key indicators obtained from the baseline and endline survey results among program participants. These indicators, which were part of the initial conceptual model and reflect program content on HTSP and family planning, include intentions to space the next birth by at least 3 years; awareness of 3 or more modern contraceptive methods; belief that contraception will “spoil” or harm one’s reproductive organs, perceived approval from a male partner for a female partner to use contraception, recent partner communication about contraceptive use, perceived joint decision making about using a method of contraception, and finally, current voluntary use of a modern method of contraception.

## PROGRAM DESCRIPTION

From 2014 to 2019, E2A and Pathfinder International worked closely with the CRS Ministry of Health and other partners to decrease the maternal mortality ratio and neonatal mortality rate and increase contraceptive use across the state through the SMGL Initiative. Using a systems approach, SMGL strengthened state, facility, and community networks to address the 3 delays that contribute to maternal mortality (delays in deciding to seek appropriate services; reaching those services; and receiving timely, quality care once the service site is accessed) and increase access to comprehensive family planning services, including long-acting reversible contraceptives, in 108 facilities in all 18 LGAs of CRS. Although the SMGL Initiative achieved reductions in facility neonatal mortality rate and facility maternal mortality ratio, the project team noted a persistent gap in reaching young women and mothers with family planning services at the community level, including those at risk of early childbearing and rapid repeat pregnancies.^24^

The SMGL Initiative noted a persistent gap in reaching young women and mothers, including those at risk of early childbearing and rapid repeat pregnancies.

Informed by evidence from formative research conducted with FTPs in 2017 (Box 1),^9^ E2A designed a program to improve voluntary modern contraceptive use and related gender outcomes among FTMs and their male partners. The FTP interventions built on existing SMGL service delivery and community platforms in 2 LGAs (Ikom and Obubra), selected on the basis of local capacity to implement community-based activities and to engage FTMs, their husbands/partners, other key influencers, and the broader community to improve contraceptive access and use. These LGAs also had sufficient numbers of adolescent and young women who were potentially FTMs. While specific data on exact numbers of FTMs in these 2 LGAs are not available, the 2015 census projections estimated that the total population of these 2 LGAs was 433,363, and approximately 9% (or 39,002) were females aged 15–24 years. While the provision of modern contraceptive methods was largely done at health facilities, the FTP interventions expanded community-based activities to deliver HTSP and family planning information, as well as counseling and referral services, and to address underlying social and gender factors that influence family planning–related communication, decision making, and action. Staff from SMGL and a local community-based organization (CBO) partner, the Greater Hands Foundation, implemented activities in 16 health facilities (a subset of public sector and faith-based facilities working with SMGL in Ikom and Obubra LGAs) and 37 communities served by these facilities. Preparations for FTP activities began in early 2018, with the main period of implementation occurring from May through August 2018.

BOX 1 Evidence to Action Project Formative Research Findings in Cross River State Nigeria

Evidence to Action conducted formative research with first-time mothers (FTMs), male partners, mothers of FTMs, and other respondents in Cross River State, Nigeria, in May 2017. The following key findings informed the design of the new first-time parent (FTP) component^9^:

- Nearly all FTMs and male partners agreed that birth spacing is beneficial for the mother, infant, and family, and could name at least one benefit of child spacing.
- Most FTMs and male partners could name or describe at least one modern family planning method, but did not know how to use any of the methods.
- Some FTPs were not sure whether family planning was safe and were concerned that its use might “spoil the womb,” thereby negatively affecting a woman’s future fertility. Married FTMs generally thought family planning was safe and beneficial, while unmarried FTMs (especially those that had never used contraception) were less likely to believe that family planning is safe for young mothers to space their children.
- Several men mentioned that they prefer “the local method” of spacing (extended postpartum abstinence), and a few men mentioned that family planning is only appropriate for women who have finished childbearing or for women in school so that they can “concentrate on their studies.” Despite this apprehension about the safety of family planning, most reported that they would approve of their wives/partners using family planning if they wanted to do so to space their births.

The formative research findings pointed to limited awareness and use of family planning services and a need to increase awareness across study sites. Recommendations also included provision of accurate and comprehensive information on family planning methods, providing effective counseling on family planning methods and services, and encouraging spousal communication to improve family planning decision making and uptake. The findings also noted young women’s/mothers’ limited use of health facilities, highlighting the need for community-based approaches that reach young people and link them to the larger health system.

FTP interventions included peer group sessions with FTMs; small group sessions with the husbands/partners of peer group members; small group sessions with older women, typically the mothers or mothers-in-law of peer group members; home visits by Greater Hands Foundation community volunteers (CVs); community sensitization; and ongoing family planning service delivery at facilities and through mobile outreach. While the Greater Hands Foundation had already been active in these communities through SMGL, new FTP activities required increased CV capacity and engagement. E2A worked with the foundation to recruit 25 certified, but not yet employed, community health extension workers (CHEWs), a health worker cadre with 2–3 years of training and typically based in peripheral health facilities. Recruited CHEWs lived in FTP intervention communities and were awaiting their official Ministry of Health posting. These CHEWs (21 women and 4 men) served as CVs dedicated to reaching FTPs and received a monthly stipend (20,000 *naira*, or approximately US$55 at the time of the intervention) and transport allowance (20,000 *naira*, or approximately US$55) to conduct activities. CVs were trained by project and CBO staff on priority health issues, including danger signs and 3 delays during pregnancy, HTSP and family planning, exclusive breastfeeding, positive parenting, and gender norms and barriers to accessing services. Trainings also stressed communication and facilitation skills, as well as project roles and responsibilities (e.g., linkages with facilities, monitoring reports). CVs participated in implementing all elements of the FTP component and were the linchpin between different activities, especially in connecting FTPs and communities with health facilities. Field activities were closely monitored by project and CBO staff attending project activities to observe progress, provide supportive supervision, and assist with any troubleshooting. Three FTP interventions—FTM peer groups, small groups with husbands/partners, and home visits by CVs—were particularly important for improving family planning–related knowledge, attitudes, gender dynamics, and actions and for increasing access to family planning services.^9^

Among FTP interventions, FTM peer groups, small groups with husbands/partners, and home visits by CVs were particularly important for family planning.

### FTM Peer Groups

The core FTP intervention was a small group activity with FTMs, grounded in the concept of creating safe spaces, peer networks, and role models for young women going through similar life experiences.^25^ Fifty groups were established in May 2018, each led by a young FTM peer leader and composed of 12–15 members. Groups met weekly in their communities for 14 sessions over the 4-month intervention period. At each 1-hour session, the peer leader used an activity card to guide discussions on a specific health or gender topic, such as HTSP, a modern contraceptive method, or problem solving within relationships (Box 2). CVs generally attended all sessions to support peer leaders, answer questions, and schedule home visits. In total, 599 out of 607 peer group members attended at least 12 of the 14 sessions.

BOX 2 Topics Addressed in First-Time Mother (FTM) Peer Groups, Cross River State Nigeria

Evidence to Action (E2A) adapted 12 activity cards from a toolkit developed by the Gender Roles, Equality, and Transformations (GREAT) project, led by the Institute for Reproductive Health of Georgetown University and implemented by Pathfinder International and Save the Children in Northern Uganda.^26^ E2A developed 2 additional cards, one on exclusive breastfeeding and the other on positive parenting. Topics included the following:

- Healthy timing and spacing of pregnancy
- Problem solving in intimate relationships
- Life aspirations
- Contraceptive methods: implants, injectables, oral contraceptive pills, condoms, emergency contraception
- Gender norms
- Communication and decision making among couples
- Desired family size
- Gender-based and intimate partner violence
- Exclusive breastfeeding
- Positive parenting

### Small Group Sessions With Male Partners

The FTP program prioritized a structured intervention with the male partners of FTM peer group members, given their influence over health decisions, including family planning use, and their own needs. By design, the male partner intervention began after the FTM peer groups, giving FTMs time to determine if they wanted to include their husband/partners. Once identified by the FTMs, CVs and “male motivators” (the partners of FTM peer group leaders) invited husbands/male partners to the small groups. This peer-to-peer approach worked well, as men were comfortable discussing the proposed activity with other men and also appreciated knowing someone who would be in the group. In total, 20 male partner groups formed in July 2018, engaging 241 men, against a target of 200, in 6 weekly sessions. These sessions explored similar health- and gender-related topics as the FTM peer groups, including HTSP, modern contraceptive methods, gender norms/roles, fatherhood, and healthy relationships. Both male and female CVs led these sessions, as their status as community resource persons helped overcome any inhibitions felt by male participants. Almost all men (231 of 241) attended the full set of sessions.

### Household Visits by CVs

Under SMGL, CVs conducted home visits during pregnancy, immediately after delivery, and 6 weeks postpartum to support maternal and infant health outcomes. The FTP component supported home visits further into the extended postpartum period for FTM peer group members. CV visits focused primarily on HTSP/family planning information, counseling, and referral services, but also addressed other pre- or postnatal issues as relevant. CVs conducted 4–6 home visits with each peer group member from May to August 2018, often at the request of the FTM or male partner, or in follow-up to an earlier conversation or referral. As much as possible, CVs made an effort to engage male partners, older women, and other household members, and often helped to address different or conflicting perspectives on possible health actions. Home visits accounted for the majority of family planning referrals given and completed. The multiple points of contact over the 4-month intervention period proved instrumental in building FTPs’ trust and confidence in CVs and, importantly, creating linkages with the broader health system.

## METHODOLOGY

This evaluation employed a quantitative pretest-posttest design with program participants to evaluate outcomes related to knowledge, attitudes, and behaviors on family planning and HTSP, exclusive breastfeeding, child development and parenting, and gender-equitable relationships between FTMs and their male partners. All data collection tools were piloted for suitability, reliability, coherence, and clarity; corrections were made as needed. Baseline and endline structured interviews were carried out using precoded questionnaires administered to FTMs and their male partners who were members/participants of intervention groups (small group sessions), before and after participation in these groups.

The evaluation focused on knowledge, attitudes, and behaviors on family planning and HTSP, exclusive breastfeeding, child development and parenting, and gender-equitable relationships.

### Sample Size

Using a sample size calculation, the program team determined that a sample of 300 FTM peer group members and 200 male partner group members would be sufficient to detect a 10-percentage-point increase in current use of family planning (a key program outcome indicator) from an assumed baseline value of or near zero. This would yield a sample detecting a significant difference from baseline to endline at the *P*<.05 level of significance with a design effect of 2.0. A 2-stage (peer groups and individual members) cluster sampling scheme was used to sample FTM respondents. The research team proportionately allocated the FTM sample (N=300) among each of the 2 LGAs based on the total number of participants and peer groups in each LGA. The study team randomly sampled respondents at both baseline and again at endline from the same 32 FTM peer groups. Due to the smaller size of the male partner program, a research team interviewed all male partners participating in the program from each of the 20 male partner groups at baseline and endline. The final achieved sample size was 338 FTMs at baseline, 339 FTMs at endline, 245 male partners at baseline, and 225 male partners at endline (Table 1).

**TABLE 1.** Criteria for Selection of Respondents and Achieved Sample Size Among Young First-Time Parents, Cross River State, Nigeria

**Selected Participants**	**Baseline**		**Endline**	
	**Ikom**	**Obubra**	**Ikom**	**Obubra**
First-time mothers At least 10 FTM members were randomly sampled at both baseline and endline from each of the selected peer groups in both LGAs	15 of 24 peer groups randomly selected at baseline; 150 FTMs randomly selected from each of the 15 groups	17 of 26 peer groups randomly selected at baseline; 188 FTMs randomly selected from each of the 17 groups	The same 15 peer groups selected at baseline were interviewed at endline; 149 FTMs randomly selected from each of the 15 groups	The same 17 peer groups selected at baseline were interviewed at endline; 190 FTMs randomly selected from each of the 17 groups
Male partners of FTMs All male partners participating in peer groups in both LGAs were selected for the study and interviewed	All (census) 10 peer groups selected at baseline; all members of each group interviewed at baseline; 123 male partners interviewed	All (census) 10 peer groups selected at baseline; all members of each group interviewed at baseline; 122 male partners interviewed	All (census) 10 peer groups selected at baseline; all members of each group interviewed at endline; 114 male partners interviewed	All (census) 10 peer groups selected at baseline; all members of each group interviewed at endline; 111 male partners interviewed

Abbreviations: FTM, first-time mother; LGA, local government area.

### Ethical Review

The study protocol and other required documents were submitted to the Government of CRS of Nigeria Health Research Ethics Committee (CRS-HREC) in Calabar, Nigeria, and to PATH’s research determination committee (RDC) in the United States in late 2017. E2A and Pathfinder International received approval to proceed with the research from the CRS-HREC on March 2, 2018. On February 26, 2018, PATH’s RDC approved the application and determined it to be “not research,” therefore obviating the need for any additional U.S.-based institutional review board review, including PATH/US institutional review board.

### Data Collection

Baseline data collection took place May 9–18, 2018, for FTMs and July 9–15, 2018, for male partners, and endline data collection for both FTMs and male partners took place from August 20 to September 2, 2018. At baseline, interviews took place during the initial group activities; a trained research team conducted private, one-on-one interviews with recruited FTMs and male partners/fathers who agreed to enroll in their respective group-based activities and consented to participate in the study. At endline, participants were recruited for private one-on-one interviews at the conclusion of the final group session. The research team of field-based staff conducted face-to-face structured interviews using standardized, precoded questionnaires at both baseline and endline. For all interviews, participants received a summary of the study and were requested to sign a consent form (with provisions for thumbprint signatures). Signed consent was obtained and a copy given to participants. Interviews were conducted in either English or Pidgin language.

### Data Management and Analysis

The research team collected data using Android-based mobile phones with the Open Data Kit application. The mobile phone data entry application included built-in consistency checks and skips. The research team uploaded the dataset to a platform storage server, where it was monitored centrally during the period of field data collection. The team then downloaded the dataset to Excel and cleaned, labeled, and checked it for inconsistencies.

SPSS Version 22 was used to perform a descriptive data analysis, using simple frequencies and bivariate analyses. Based on the sampling scheme, the baseline and endline FTM samples (which were randomly generated at both times) were treated as independent samples, and the male partner samples were treated as nonindependent repeated measures. Although FTMs were randomly recruited at baseline and endline, the peer groups to which they belonged were the same at baseline and endline. Therefore, the authors of this study conducted a post hoc analysis of independence between the 2 FTM samples and determined that about 75% of the sample at baseline was included again at endline. A sensitivity test was therefore conducted with only the repeated measures subset to confirm the robustness of the analysis and the statistical significance of the FTM findings. All statistical comparisons of the FTM data presented in this paper were reanalyzed as repeated measures using McNemar’s test of significance for categorical data and the paired t-test for continuous data, both with and without complex sampling (based on 2-stage cluster sampling), using the subsample of participants measured at both baseline and endline. Significance levels of these findings did not vary from the analysis of independent samples. Therefore, 1-tailed Pearson chi-square tests for categorical data and analysis of variance F-tests for continuous data were used to present the statistical significance of differences between baseline and endline and other variables of interest among FTMs. In addition, a logistic regression analysis of current use of modern contraceptive methods by FTMs was also performed to determine if a significant change took place over the course of the interventions in the uptake of contraception after controlling for key demographic variables, partner characteristics, couple communication, and attitudes.

A different analytical approach was used for the male partner sample, as it was a census of all program participants (and thus the samples were not independent). The unmatched sample was dropped (n=21), and the McNemar’s test and paired t-test were used to present statistical differences at baseline and endline using the matched sample (n=224). In addition, all statistical comparisons of the male partner data presented in this paper were analyzed using these tests with and without complex sampling (based on 2-stage cluster sampling). Significance levels of these findings did not change based on adjusting for 2-stage cluster sampling, remaining highly significant.

## RESULTS

### Sociodemographic Characteristics

The baseline and endline survey data provided useful information about the characteristics of FTMs and a subset of their male partners who joined and stayed engaged in interventions. While some recruitment inclusion criteria were set for FTM peer group members (under 25 years, pregnant or with first child) and their male partners (identified and nominated by interested/willing FTM participants), activities were otherwise open to FTMs and male partners who wanted to participate. Table 2 presents select background characteristics of FTMs and a nominated subset of male partners engaged in the FTP interventions. Almost all FTMs were within the required age limit at baseline, with roughly 63% aged 20–24 years and 29% aged 15–19 years. Participating male partners were most likely to be older; 30% of male partners were aged 30 years or older at baseline and endline.

**TABLE 2.** Percentage Distribution of Age, Marital Status, Local Government Area, and Education Level by Participant Group and Baseline/Endline Among Young First-Time Parents, Cross River State, Nigeria

	**First-Time Mothers**		**Male Partners**	
	Baseline (n=338)	Endline (n=339)	Baseline (n=224)	Endline (n=224)
Age, %				
15–19 years	28.7	28.3^a^	1.3	0.9
20–24 years	62.7	67.8^a^	28.6	30.4
25–29 years	1.2	2.7^a^	40.2	38.8
30 years plus	0.0	0.3^a^	29.9	29.9
Don’t know/missing	7.4	0.9^a^	0.0	0.0
Mean age, years	20.6	21.1^b^	27.5	27.4
Local government area, %				
Ikom	44.4	44.0	50.9	50.9
Obubra	55.6	56.0	49.1	49.1
Marital status, %				
Never married	62.7	53.1^b^	30.4	31.7
Living with partner/married	37.3	45.4^b^	69.6	68.3
Divorced/separated/widowed	0.0	1.5^b^	0.0	0.0
No. of living children, %				
0	14.5	7.7^b^	9.8	4.5
1	85.5	92.0^b^	85.3	90.2
2	0.0	0.3^b^	4.9	5.4
Age of youngest child (among participants with at least 1 child)				
Mean age of youngest child (months)	6.9 months (n=289)	8.7^a^ (n=312)	8.8 months (n=199)	10.9 months (n=214)
Residential arrangement, %				
Currently lives with partner	45.0	43.4	75.4	72.8
Education level, %				
Primary	13.9	10.9	3.6	4.0
Junior Secondary (completed)	35.2	36.6	9.8	10.3
Secondary (completed)	47.6	45.4	67.9	67.0
Polytechnic	1.8	2.9	4.0	3.1
University	1.5	4.1	14.7	15.6
Works to earn money, %				
Yes	36.1	56.9^a^	84.8	86.6

a Chi-square *P*<.000.

b Chi-square *P*<.05.

At baseline, most FTMs reported that they were not married/living with their partner (63%, N=338), and 68% of a subset of nominated male partners (N=245) reported that they were either married or living with their partner. The majority of FTM participants (86%) had 1 child with a mean age of 6.9 months at baseline, with another 14% pregnant with their first child. The data also show that most (90%) male partners enrolled in the program were also first-time fathers. A majority of both FTMs and male partners who participated in the FTM program reported completing a secondary or higher level of education. Although most (85%) male partners reported being currently employed at baseline, only about one-third of FTMs (36%) reported working at baseline, likely due to their recent pregnancy and delivery.

Between baseline and endline, FTMs had a few significant differences in some of these variables. FTMs at endline were slightly older (21.1 years of age) than at baseline (20.6 years of age), as were their babies, largely due to the 4-month interval between data collection efforts for FTMs. (Only 2 months elapsed between data collection efforts for male partners.) In addition, by endline, FTMs were more likely to be in union or married and were more likely to be working to earn money than at baseline. As expected, no significant differences were noted among male partners from baseline to endline with respect to these key demographic variables.

### Birth Spacing Intentions

**FIGURE 1.**
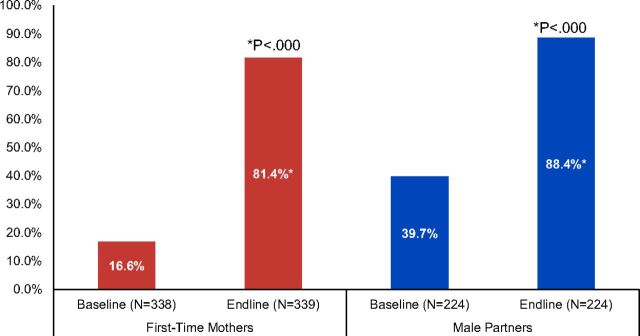
Percentage of First-Time Parents Who Do Not Want Another Child or Who Wish to Wait 3 Years or Longer to Have Their Next Child, Cross River State, Nigeria

One of the key messages of the FTP interventions was to encourage a spacing gap of 3 years or more between births. Figure 1 shows that at baseline, only 17% of FTMs and 40% of male partners at baseline preferred no more children or wished to wait 3 years or longer to have another child. At endline, 81% of FTMs (*P*<.000, Pearson chi square) and 88% of male partners preferred no more children or to wait 3 years or longer (*P*<.000, McNemar’s test). Importantly, an alignment in birth spacing intentions generally occurred for both FTMs and male partners.

One of the key messages of the FTP interventions was to encourage a spacing gap of 3 years or more between births.

### Awareness of Modern Contraceptive Methods

**FIGURE 2.**
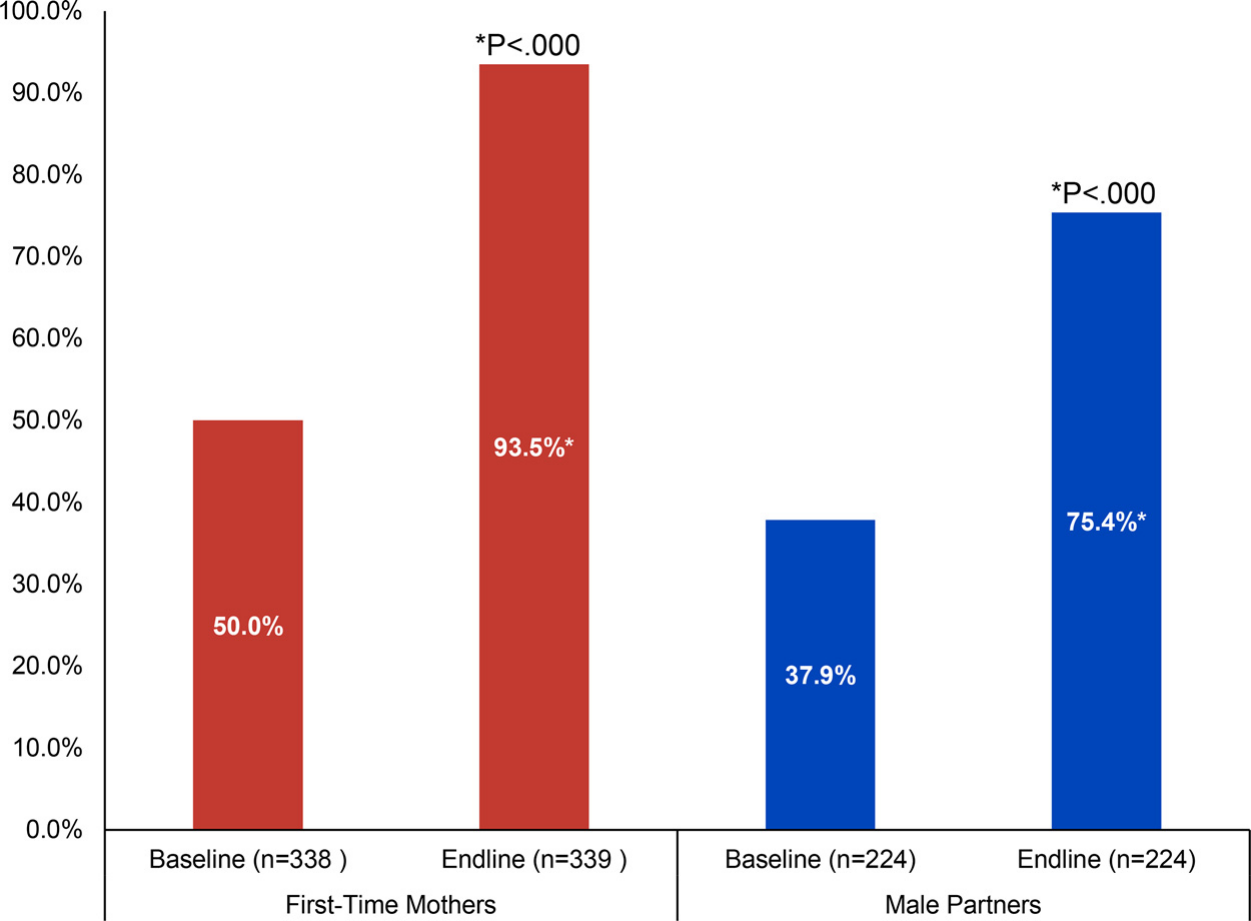
Percentage of First-Time Parents Who Can Name at Least 3 Modern Contraceptive Methods, Cross River State, Nigeria

The FTM and male partner interventions emphasized knowledge and use of postpartum contraception. Knowledge of modern contraceptive methods increased over the course of the interventions, with the percentage of FTMs and male partners who could spontaneously recall at least 3 modern methods increasing significantly (see Figure 2). The percentage of FTMs and male partners who could spontaneously recall at least 3 modern methods nearly doubled over the life of the interventions, increasing significantly among FTMs from 50% at baseline to 94% at endline (*P*<.000, Pearson chi-square), and among male partners from 38% at baseline to 75% at endline (*P*<.000, McNemar’s test).

### Myths and Misperceptions of Using Modern Contraception

**FIGURE 3.**
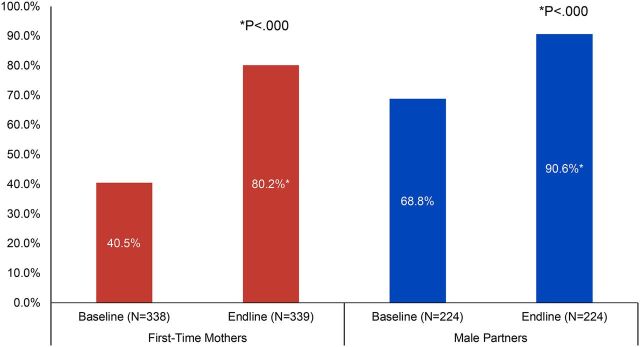
Percentage of First-Time Parents Who Agree That Using Contraceptives May Negatively Affect One’s Ability to Have Children in the Future, Cross River State, Nigeria

A key finding from formative research conducted prior to the FTP interventions was that many FTMs and their male partners believed that using contraception can damage a woman’s reproductive organs and create difficulties in conceiving or can even cause permanent sterility after discontinuation. Thus, most believed that it is best for a woman to use contraception for limiting fertility only after achieving one’s desired family size. Correcting this misconception was an area of focus throughout the interventions. Figure 3 presents the percentage of interviewed participants who held this belief at baseline and endline. At baseline, 55% of FTMs and 29% of male partners agreed that using contraception could negatively affect a woman’s ability to have children in the future. At endline, only 1% of FTMs (*P*<.000, Pearson chi-square) and 7% of male partners held this belief (*P*<.000, McNemar’s test).

### Spousal/Partner Approval for Using Modern Contraception

Husband’s or partner’s approval for using a method of contraception (or perceived approval by FTMs) may be a critical factor in facilitating an FTM who is married or in union to accept and use contraception. FTMs were asked if they thought that their husband/partner would approve if they wanted to use a method of contraception to space their next child, and male partners were asked if they themselves would approve of their wife/partner using a method of family planning, as well as whether or not they would give her money to seek family planning services. At baseline, only about two-thirds (67%) of the FTM participants thought that their partner would approve of their use of family planning to space their next child, which increased significantly to 80% (*P*<.000, Pearson chi-square test) at endline (see Table 3). Male partners, however, were much more likely to approve at baseline (91%), and this did not change significantly at endline (94%). Nearly all (94%) of male partners agreed that they would be willing to support their female partner/wife with money to seek family planning services at endline, which significantly increased from baseline (88%, *P*<.05, McNemar’s test).

**TABLE 3.** Percentage Distribution of Partner Support for Family Planning by Participant Group and Baseline/Endline

**Variable**	**First-Time Mothers**		**Male Partners**	
	Baseline, %(n=338)	Endline, % (n=339)	Baseline, % (n=224)	Endline, % (n=224)
Agrees that husband/partner would approve of using family planning to space next child	66.9	79.6^a^	90.6	93.8
Would give wife/partner money to seek services if she wanted to use family planning to space her next birth			87.9	93.8^b^

a Chi-square *P*<.000.

b McNemar’s test *P*<.05.

### Couple Communication on Family Planning

**FIGURE 4.**
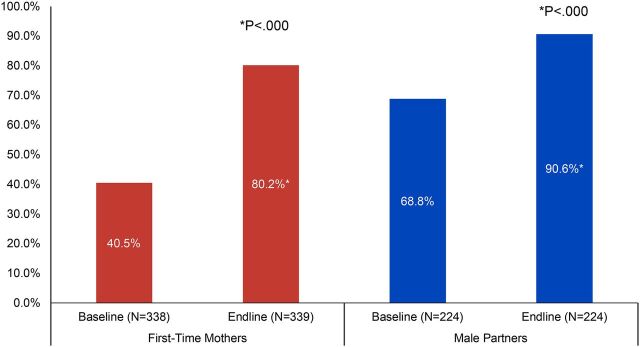
Percentage of First-Time Parents Who Have Discussed Family Planning With Their Partner as a Way to Space Children in Past 3 Months, Cross River State, Nigeria

Having discussions with one’s partner or other influential people is often associated with interest in and voluntary use of family planning. The FTP interventions included activities and discussion around partner communication on family planning and birth spacing. Figure 4 presents baseline and endline data on discussions about family planning with partners and other influencers among both FTMs and male partners. Reported discussions about family planning among FTMs (regardless of marital/union status) doubled from baseline (41%) to endline (80%, *P*<.000, Pearson chi square) and increased significantly among male partners from 69% to 91% (*P*<.000, McNemar’s test). Discussions among FTMs and male partners with other influential people also increased from baseline to endline (data not shown), from 28% to 55% for FTMs (*P*<.000) and from 17% to 42% for male partners (*P*<.000, McNemar’s test). When asked with whom they discussed family planning in the past 3 months, FTMs were most likely to report discussing family planning with a mother (43%), sister (34%), or friend (51%) at endline (n=187, data not shown); male partners were most likely to discuss family planning with a friend (73%) or a brother (21%) at endline (n=95, data not shown).

Having discussions with one’s partner or other influential people is often associated with interest in and voluntary use of family planning.

### Couple Decision Making About Family Planning

Both FTMs and male partners were asked at baseline and endline about decision-making responsibility for using family planning (data not shown). The percentage of FTMs and male partners who reported that they should decide together to use family planning was high even at baseline: 82% (N=338) of FTMs and 88% (n=224) of male partners agreed that using family planning should be a joint decision before the intervention. However, this percentage significantly increased for both participant groups by endline; by the end of the intervention, 96% (N=339; *P*<.000, Pearson chi-square) of FTMs and 99% (N=224; *P*<.000, McNemar’s test) of male partners agreed that using family planning should be a joint decision. Perhaps even more important, relatively few FTMs reported that husbands/partners were the primary decision maker about family planning, suggesting that contraceptive use was largely voluntary for these young women.

### Current Voluntary Use of Modern Contraception

**FIGURE 5.**
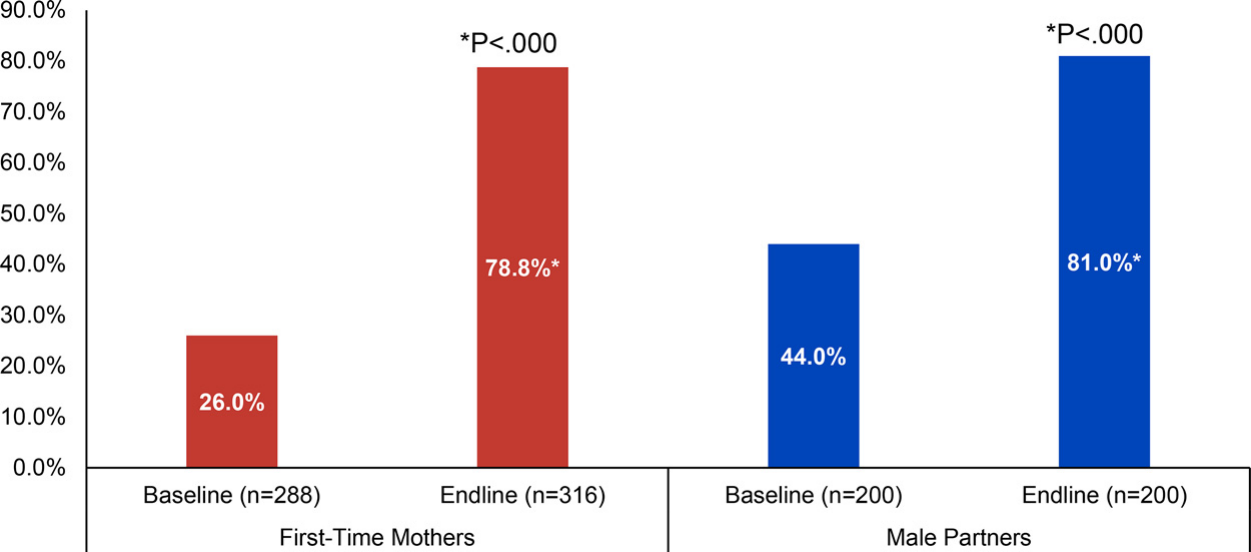
Current Use of a Modern Contraceptive Method (Among First-Time Parents Not Currently Pregnant), Cross River State, Nigeria

The key objective of the FTP interventions was to increase current voluntary use of a modern contraceptive method. Figure 5 shows that current use of a contraceptive method among both FTMs and male partners significantly increased from baseline to endline. Current use increased from 26% (n=288) to 79% (n=316) among nonpregnant FTMs (*P*<.000, Pearson chi-square), and from 44% to 81% (n=200) among male partners (*P*<.000, McNemar’s test). Importantly, other positive changes in family planning knowledge, attitudes, communication, and decision making all support the overall increase in informed, voluntary contraceptive use by FTP participants.

A logistic regression analysis was also performed to confirm the bivariate findings above, predicting current use of any modern family planning method (implants, intrauterine devices, injectables, oral pills, male or female condoms, emergency contraception, or standard days method) among both FTMs and male partners (in separate models, data not shown). All relevant demographic variables were included in the model, as well as attitudes toward family planning, couples’ discussions about family planning, perceived partner approval and joint decision making for family planning, as well as a variable representing the survey wave (baseline/endline, with baseline as the reference category). This analysis revealed that for both FTMs and male partners, survey wave was highly significant (*P*<.001) with adjusted odds ratios of 3.3 for FTMs and 3.7 for male partners. This means that modern contraceptive uptake significantly increased from baseline to endline for both groups of participants. In other words, after controlling for hypothesized predictors of family planning use, including demographic factors (age, marital status, education level, age of youngest child) and all attitudes related to family planning use presented in this report (including perceived safety of contraceptive methods, partner approval, and decision making related to family planning use), FTMs were approximately 3 times more likely and male partners nearly 4 times more likely to be using a modern family planning method at endline, compared with baseline.

The key objective of the FTP interventions was to increase current voluntary use of a modern contraceptive method.

### Method of Contraception Used

**FIGURE 6.**
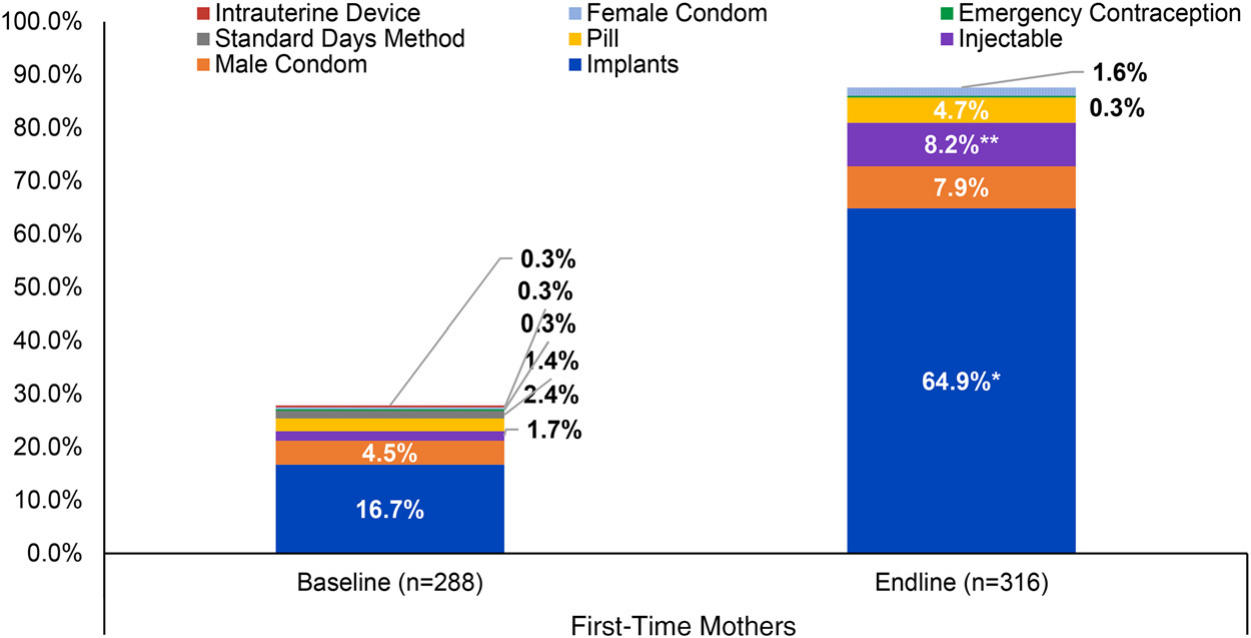
Current Modern Contraception Method Used Among First-Time Mothers Not Currently Pregnant (Multiple Responses Possible), Cross River State, Nigeria * *P*<.000. ** *P*<.01.

Figure 6 shows the type of contraceptive method used among FTM respondents not pregnant at the time of data collection (multiple responses were possible). The graph reveals that use of implants and injectables increased significantly from baseline to endline for nonpregnant FTMs, with implants being the most commonly used method among all respondents (men’s reported use of implants also increased significantly from baseline to endline, but since nearly all male partners had an FTM partner in the program, this information is presented for FTMs only). At baseline, only 17% of FTMs (n=287) reported using an implant, whereas 65% of FTMs (n=316) were using implants at endline (*P*<.000, Pearson chi-square test). Importantly, use of implants aligns with the overall spacing intentions (majority reported 3 or more years) indicated by both FTMs and male partners at endline.

## DISCUSSION

This article has highlighted selected results of a program designed to address some of the critical barriers faced by young FTMs and their male partners in using family planning to space their second and subsequent children. Implemented by local organizations and resource persons, the interventions aimed to increase HTSP awareness and intentions, build awareness of modern contraceptive methods, dispel key myths and misperceptions about family planning, address gender norms and barriers, increase social and partner support for family planning use, and provide referrals and facility linkages for obtaining specific contraceptive methods. We examined key HTSP and family planning indicators among participating FTMs and their male partners to determine if they successfully changed attitudes and behaviors related to birth spacing and voluntary contraceptive use over the course of the program.

This intervention evaluation included a coordinated baseline and endline questionnaire among a scientific and robust sample of FTM participants and a census of male participants using a trained team of interviewers and digital mobile data collection tools. The results show that the interventions attracted and retained a diverse range of FTMs and male partners (in terms of key sociodemographic variables such as marital status and education) and was successful in improving birth spacing intentions and current use of contraceptive methods from baseline to endline, even after controlling for key sociodemographic and attitudinal variables. Important indicators of contraceptive awareness, attitudes, and couples’ communication increased significantly from baseline to endline, and also significantly predicted current use of a modern contraceptive method over the course of the intervention. Along with overall increased contraceptive uptake, FTMs and their partners chose to use more effective and/or long-acting methods (injectables and implants), perhaps reflecting, in part, their longer birth spacing preferences, interest in a newly introduced method (implant), as well as the recent increased availability of these contraceptives locally.

The interventions were successful in improving birth spacing intentions and current use of contraceptive methods from baseline to endline.

These positive findings should be interpreted with several limitations in mind. This study relied on self-reported information gathered during face-to-face interviews, subject to both courtesy and recall bias. This bias was minimized through training of interviewers and design of the questionnaire. Another limitation is related to the possibility that other family planning activities took place in CRS, Nigeria, in the same or nearby geographic areas, which also may have resulted in increased health knowledge and behaviors including a high level of family planning uptake by program participants; however, Pathfinder International’s CRS program staff reported that no other family planning-related partner activities had taken place concurrently in Ikom or Obubra LGAs. In addition, participants might have changed between the pretest and the posttest regardless of the interventions because they are maturing and learning, especially as parents of young infants. This limitation was minimized by ensuring that baseline and endline data collection coincided tightly with program implementation. A final limitation is related to the self-selection of program participants and propensity of more empowered individuals (as opposed to those in the general population) to participate. While it is highly likely that participants had some propensity toward the information and messages received during the program, baseline attitudes and knowledge were consistent with findings from the formative research (described previously).

While previous E2A FTP projects primarily focused on activities with FTMs related to HTSP/family planning, the interventions in CRS engaged male partners more systematically. We included group activities with a subset of nominated male partners of FTM peer group members to address gender norms, increase male engagement in HTSP and family planning, and promote couple communication and joint decision making. Specific activities were included to generate evidence on both the implementation experience and on health and family planning outcomes for FTMs and their male partners emerging from this programming effort. Importantly, endline results show that FTMs and their partners were generally aligned on key family planning attitudes and birth spacing intentions, which may have facilitated increased contraceptive use and method choice. These results suggest that couple-oriented interventions or joint activities can work well—even in a context in which many FTPs are not in formal unions or necessarily living in the same household.

Our experience in CRS suggests that FTMs and male partners may be particularly open to HTSP and family planning use because they face the practical and financial realities of raising a child. Community-based resources like CBOs, CVs (or similar community health workers), peer leaders, and others provided FTPs with tailored and timely access to information and services, as well as linkages with health facilities critical to ensuring access to a full range of needed services. Many FTMs do not routinely access health facilities or may not be ready to consider family planning options when they do. Therefore, such approaches may work better than only integrating family planning into clinic-based services (e.g., postpartum family planning, postabortion care services, or even family planning integration into antenatal care), especially where there are inequities in access to and use of health care by young FTMs.

While our project included multiple interventions focused on FTPs, all activities were implemented over a 4-month period through existing health facilities and a local CBO, using trained and certified (but not yet employed) CHEWs. Several elements, such as home visits, community outreach, and the provision of modern contraceptive methods, were already included within the general mandate of the primary health care system. Building on existing community- and facility-based resources to identify and reach FTPs with tailored activities generated compelling results and provided a model that can be adapted based on available resources and scaled-up across the state.

## CONCLUSION

The E2A experience in CRS shows that tailored interventions with FTPs can achieve important HTSP and family planning results within a relatively short time frame. FTMs and partners are coping with multiple challenges as new parents and are receptive to information and options that allow them to delay subsequent births. The emerging high demand for family planning across diverse FTMs and partners—especially for more effective and longer-acting contraceptive methods—underscores the importance of engaging FTPs during this critical moment in their reproductive lives. In particular, the CRS experience suggests 3 essential program elements: (1) ensuring the availability of modern contraceptive methods (especially implants) through local health facilities; (2) using locally based resource persons or community-based health workers to conduct home visits with FTPs to provide tailored health information and referrals, as well as build linkages with the formal health sector; and (3) using activities that address gender norms and couple dynamics to foster better alignment, communication, and joint action on reproductive issues. All activities can be implemented through locally based resource persons, who are best positioned to identify and reach young FTPs of different characteristics and situations. The results that can be achieved, along with high levels of engagement from FTPs, demonstrate the importance of investing in these types of interventions, ideally addressing all priorities for family planning, reproductive health, and maternal, neonatal, and child health across the FTP lifestage, from pregnancy through the postpartum period.

## Notes

**Peer Reviewed**

**First published online:** November 19, 2020
